# The Beneficial and Adverse Effects of Autophagic Response to Caloric Restriction and Fasting

**DOI:** 10.1016/j.advnut.2023.07.006

**Published:** 2023-07-30

**Authors:** Roya Shabkhizan, Sanya Haiaty, Marziyeh Sadat Moslehian, Ahad Bazmani, Fatemeh Sadeghsoltani, Hesam Saghaei Bagheri, Reza Rahbarghazi, Ebrahim Sakhinia

**Affiliations:** 1 Infectious and Tropical Diseases Research Center, Tabriz University of Medical Sciences, Tabriz, Iran; 2 Department of Medical Genetics, Faculty of Medicine, Tabriz University of Medical Sciences, Tabriz, Iran; 3 Student Committee Research Center, Tabriz University of Medical Sciences, Tabriz, Iran; 4 Drug Applied Research Center, Tabriz University of Medical Sciences, Tabriz, Iran; 5 Stem Cell Research Center, Tabriz University of Medical Sciences, Tabriz, Iran; 6 Department of Applied Cell Sciences, Advanced Faculty of Medical Sciences, Tabriz University of Medical Sciences, Tabriz, Iran

**Keywords:** short-term and long-term, calorie restriction, autophagy, cellular homeostasis, therapeutic effects

## Abstract

Each cell is equipped with a conserved housekeeping mechanism, known as autophagy, to recycle exhausted materials and dispose of injured organelles via lysosomal degradation. Autophagy is an early-stage cellular response to stress stimuli in both physiological and pathological situations. It is thought that the promotion of autophagy flux prevents host cells from subsequent injuries by removing damaged organelles and misfolded proteins. As a correlate, the modulation of autophagy is suggested as a therapeutic approach in diverse pathological conditions. Accumulated evidence suggests that intermittent fasting or calorie restriction can lead to the induction of adaptive autophagy and increase longevity of eukaryotic cells. However, prolonged calorie restriction with excessive autophagy response is harmful and can stimulate a type II autophagic cell death. Despite the existence of a close relationship between calorie deprivation and autophagic response in different cell types, the precise molecular mechanisms associated with this phenomenon remain unclear. Here, we aimed to highlight the possible effects of prolonged and short-term calorie restriction on autophagic response and cell homeostasis.

**Statement of Significance**

This review article presents the evidence supporting the modulatory effects of calorie restriction on autophagic flux under physiological and pathological conditions. Both adaptive (useful) and excessive (harmful) autophagic responses can be stimulated, depending on the metabolic status, by short- and long-term diet programs. Attention should be taken to the application of calorie restriction along with therapeutic protocols under pathological conditions.

## Introduction

The term autophagy refers to cellular mechanisms associated with self-eating and diverse homeostatic phenomena. Activation of autophagy helps the host cells to eliminate misfolded, aggregated proteins and injured organelles [1]. Besides its role in cellular homeostasis, autophagy is actively involved in the development, differentiation, and regenerative potential of several embryonic and adult cells [2,3]. In eukaryotes, the autophagy machinery and proteasomes are the 2 main degradation systems for the elimination of digested molecular cargo [4]. In contrast to proteasomal degradation, autophagy is the only way to eliminate entire injured organelles [2]. To date, 3 different types of autophagy mechanisms, namely macroautophagy, microautophagy, and chaperone-mediated autophagy, have been indicated in eukaryotic cells [2]. Recent works have established that macroautophagy is the main autophagy mechanism in most cell types and is hereafter referred to as autophagy. From ultrastructural aspects, the autophagic response is initiated by the formation of double-membrane vesicles named autophagosomes [5]. In the latter steps, autophagosomes translocate and fuse with lysosomes for enzymatic degradation via the activity of several acidic hydrolases [6]. Compared to macroautophagy, microautophagy is thought of as a nonselective process and is initiated by the direct engulfment and sequestration of cytosolic components via the lysosomal membrane. In the last autophagy type (chaperone-mediated autophagy), a complex of chaperone proteins and target proteins are directed into the lysosomes via the activity of LAMP-2A [7].

Despite the protective role of autophagy on injured cells, abnormal or excessive autophagic responses cause several pathological outcomes via the stimulation of programmed cell death such as apoptosis, pyroptosis, etc. [[8], [9], [10], [11]]. Likewise, a severe reduction in autophagy activity can contribute to diverse pathological conditions such as genome instability, anaplastic changes, infection, premature aging, and metabolic disease [[12], [13], [14], [15], [16]]. Due to the mutual crosstalk between several autophagy constituents and other signaling cascades, pleiotropic effects have been attributed to the autophagy machinery [17,18]. The existence of complex autophagy machinery and shared molecular effectors lead to different simultaneous reaction cascades [19]. Along with these descriptions, it is logical to hypothesize that specific metabolic conditions can regulate the autophagic response either in physiological or pathological situations [20]. Molecular investigations have revealed that low energy levels or deprivation of essential nutrients like amino acids and glucose can lead to ATP depletion and increased AMP/ATP ratio, resulting in autophagy induction [21]. Under these conditions, the autophagy machinery is recalled to compensate for ATP limitation, whereas the promotion of autophagic efflux and subsequent biological events are closely linked to the consumption of ATP [22]. The exact relevance of the duration of starvation and activation of adaptive or excessive autophagy remains unknown. Here, we collected recent data associated with the possible therapeutic/detrimental effects of fasting on the autophagic response in terms of cellular homeostasis.

**FIGURE 1:**
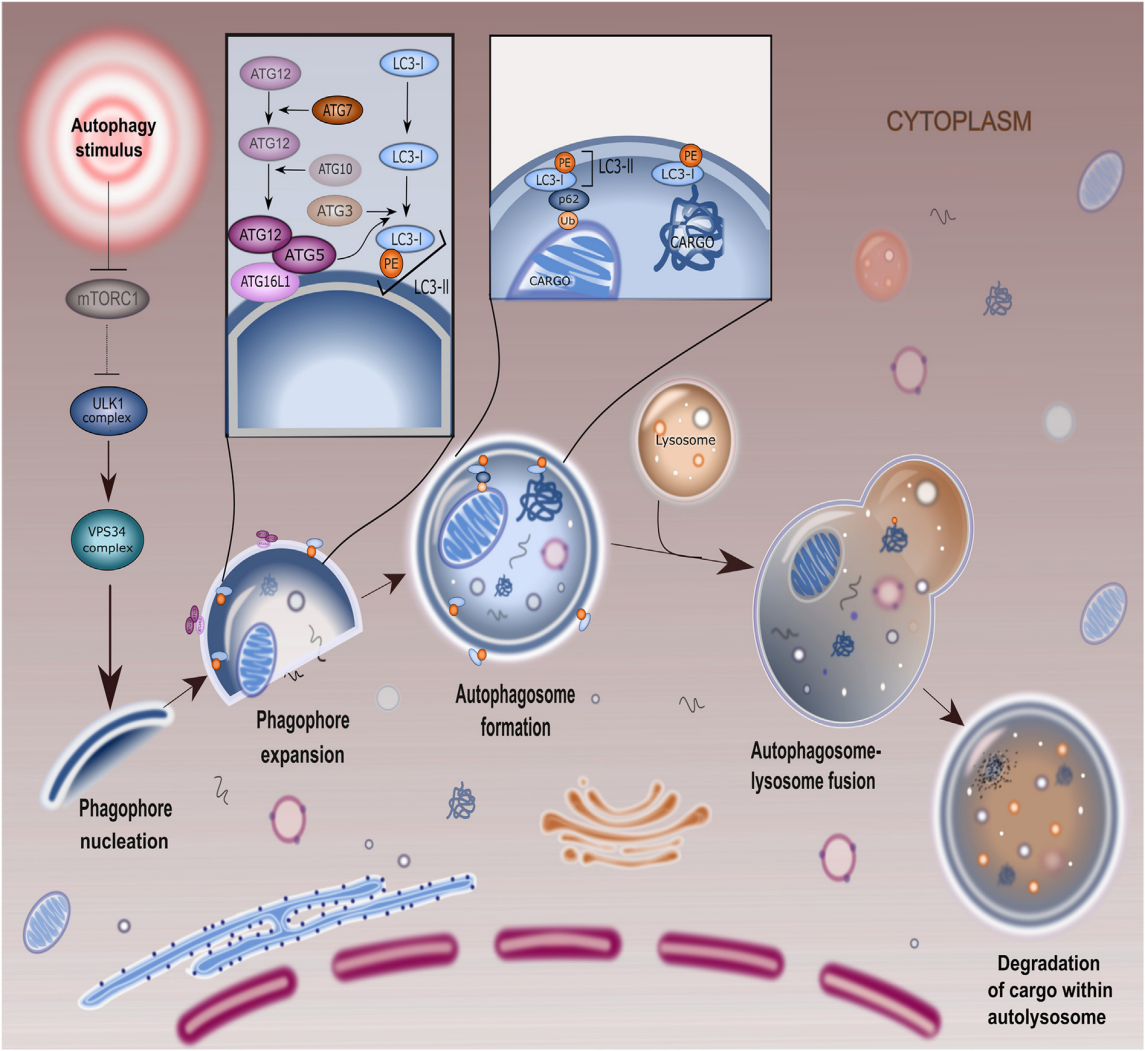
Autophagy molecular machinery. The process of autophagy consists of several consequent molecular and subcellular morphological changes. In the early steps, the activation of autophagy upstream molecules leads to phagophore formation and expansion. These units sequester the damaged organelles, aggregated proteins, etc. By the activation of several Atgs, phagophores mature into autophagosomes followed by fusion with lysosomes and the formation of autophagolysosomes. Inside the autophagolysosomes, enzymatic digestion is orchestrated via varied hydrolases, leading to massive enzymatic digestion. The digested cargo is recycled into the cytosol or protrudes out of the host cells. Atg, autophagy-related protein; mTOR, mammalian target of rapamycin.

As abovementioned, the autophagic response is initiated by the formation of double-membrane vesicles, namely autophagosomes. This biological phenomenon depends on the activity of nearly 16 Atgs and 2 distinct ubiquitin-like conjugation systems (Figure 1) [23]. Two complexes are required to promote the formation of autophagosomes. The first complex is composed of type III PI3K, Vps34, Atg14, Atg6/Beclin1, and Vps15/p150.73, and the second complex is associated with the activity of the serine/threonine kinase Atg1 [24]. In yeast, Atg8 or Atg13 and Atg17 are essential for the kinase activity of Atg1 whereas in mammals, LC3, which is an ortholog of Atg8, can promote the activity of Atg1 along with GATE-16 and GABARAP [25]. In the next step, autophagosomes become elongated via the activity of ubiquitin-like conjugation pathways. For this purpose, cells can use 2 pathways consisting of LC3/GABARAP/GATE-16 and Atg12 [26]. Due to the proteolytic activity of Atg4 on the Atg8 (carboxyl terminus), a glycine residue is exposed to generate the autophagosome [27]. After that, phosphatidyl ethanolamine is covalently attached to Atg8 (lipidated LC3-II in mammals) [28]. With the progression of autophagosome formation, the Atg16-Atg5-Atg12 complex is separated from the autophagosomal membrane, and complete autophagosomes can fuse with lysosomes [29]. The procedure is followed by the formation of autophagolysosomes in which autophagic cargo is degraded via lysosomal activity. In an alternative pathway, autophagosomes fuse with late endosomes and form amphisomes [30]. Molecular investigations have indicated that both amphisomes and autophagosomes converge eventually in lysosomes to digest and recycle their contents [31]. Several factors like Rab and Arf proteins with GTPase activity, tethering proteins like SNAREs, tethering adaptors, along with motor proteins promote the direct interaction of autophagosomes with lysosomes [31]. Autophagocytosed macromolecules are recycled into the cytosol to maintain bioenergetic homeostasis or are secreted out of the cells [32]. It should not be forgotten that the increase of autophagosomes (known also as autophagy flux) can reflect both increased autophagic response and abnormal reduction of autophagolysosome formation [33]. Therefore, the increase of intracellular autophagosomes does not necessarily reflect a complete autophagy response.

In this review article, we aimed to highlight the possible effects of prolonged and short-term calorie deprivation on autophagic response and cell homeostasis. The molecular mechanisms by which calorie restriction can mediate both adaptive and excessive autophagic responses are discussed in detail. Whether and how autophagy modulation by calorie restriction can help the host cells to circumvent insulting conditions is at the center of the debate.

### Health Benefits of Intermittent Fasting

The term intermittent fasting (IF) includes different short- and long-term diet programs with regular cycles between the time of eating and fasting [[34], [35], [36]]. Calorie-restricted programs can usually last for 24 h to 3 wk, providing 30% of total energy demands [37]. Along with several programs recommended by nutritionists, IF is complete or partial abstinence from all drinks and foods in religious disciplines [38]. For decades, different forms of IF regimes have been used in several studies [39]. In the alternate-day fasting protocol, fasting is done every other day, and the consumption of energy-generating food products is prevented, whereas during feeding time (d) the studied groups are allowed access to food ad libitum. Of note, alternate-day modified fasting is a type of alternate-day fasting in which total energy intake reaches below 25% to 30% and is done in a limited period (open eating window: 2–4 h). A modified fasting regimen is another type of IF that supports 20% to 25% of daily energy using regular fasting days. This method is based on 5:2 diet, with 2 fasting days per week and usual eating on other days. Time-restricted feeding includes specific time frames of eating and fasting. Ramadan, with time-restricted eating rules, restricts Muslims to eat from dawn to sunset. During the eating time (4–10 h), the energy amount is not limited [37,39]. Several findings are consistent with the fact that calorie restriction is linked to longevity and enhances protection against chronic pathologies by the regulation of anti-inflammatory responses [40]. IF may be an alternative method to conventional therapeutic regimes by exerting oncostatic properties and even increasing cancer patients' tolerance to chemotherapy. In support of this notion, healthy cells are more resistant to chemotherapy-induced injury; therefore, the intensity of off-target side effects is less in cancer patients who have undergone fasting [41]. To be specific, the exposure of normal cells to calorie restriction leads to the consumption of energy (ATP) for maintenance and regeneration purposes rather than proliferation. In tumor cells, the activation of several oncogenes such as the IGF-1R, Ras, and AKT/mTOR molecular pathways increases the proliferation rate and thus cell sensitivity to chemo/radiotherapy protocols [42]. Several works have provided evidence of bulk changes in metabolic status under regular starvation conditions [36]. In about the first 10 h after starvation, the stored glycogen in hepatic tissues is consumed followed by the breakdown of the fatty acids in muscles and adipose tissue. As a consequence, significant quantities of free fatty acids and amino acids are produced [36]. Importantly, IF alleviates inflammatory responses and leads to several ameliorative outcomes in individuals affected by asthma, rheumatoid arthritis, etc. [40]. An example is the production of IL-4, -5, and -13 by type 2 CD4^+^ lymphocytes to increase macrophage polarization toward M2 phenotypes, resulting in anti-inflammatory properties [43]. Beneficial effects of IF on gut microbiota and immune system activity may take place in patients with multiple sclerosis [44]. The molecular identity of the peripheral mononuclear cells in 21 volunteers with 24-h fasting and 3-h refeeding revealed the activation of CD4^+^ Th2 cells in healthy groups and asthmatic counterparts [45,46]. The exact relevance of fasting and immunomodulatory effects is associated with the activation of FOXO4 and its canonical target FK506-binding protein 5. These factors are thought of as fasting-response elements in terms of the immune system with the potential to regulate the production and release of inflammatory cytokines from Th1 and Th17 lymphocytes [47]. In a similar experiment, time-restricted fasting significantly reduced the number of CD16^+^ and CD56^+^ natural killer cells without any effects on circulating CD3^+^, CD4^+^, and CD8^+^ lymphocytes [48]. Fasting may increase the activity of the antioxidant system against oxidative stress. For instance, SOD, an enzyme involved in the neutralization of ROS, is stimulated in cells subjected to IF [49]. Data indicated that the absolute protein concentrations of soluble intracellular adhesion molecule-1, LDL, and T3 hormone are reduced in middle-aged populations subjected to short- and long-term alternate-day fasting [50]. Biochemical studies demonstrated that 49 metabolites exhibited a 20% reduction and these values were ∼44.9% in amino acids. The apparent reduction of certain amino acids like methionine, a pro-aging factor, can postpone the aging procedure [51]. Another antiaging benefit of IF is related to the regulation of stem cell dynamic growth and function [52,53]. These cells can restore the function of damaged cells and replace lost cells. The conduction of fasting programs in animal models can lead to the elimination of age-related changes like AD and accelerated healing capacity under ischemic stroke [40]. Based on the histological examination, the proliferation of rodent hippocampus neural stem cells is increased ∼3 wk after IF. These changes are consistent with the local distribution of BDNF [54]. Of note, stimulation of the mTOR axis reduces telomere shortening and maintains pluripotency potential under IF conditions [55,56].

## IF and Autophagy

### Mechanisms of IF-induced autophagy

**FIGURE 2:**
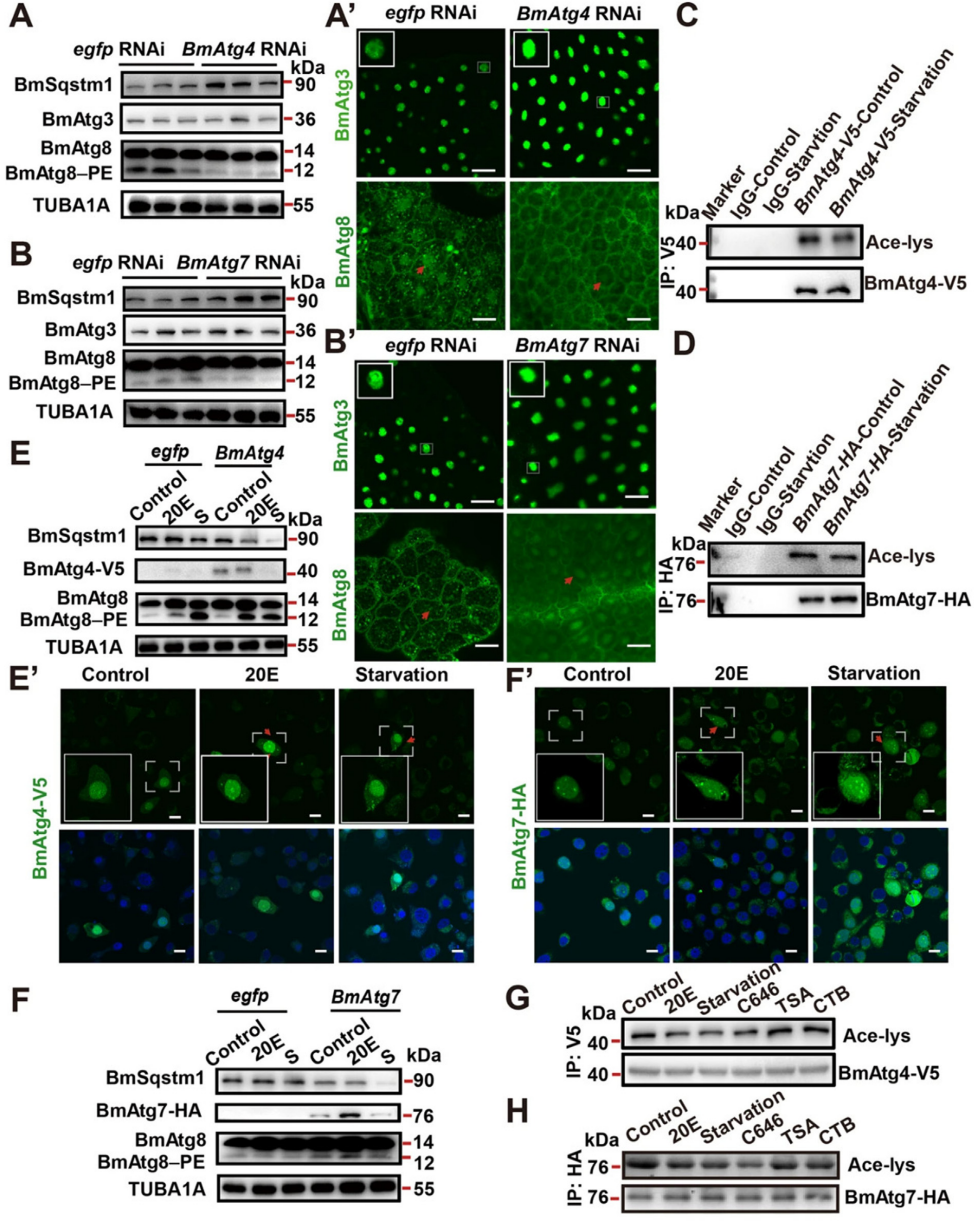
Monitoring protein concentrations of BmSqstm1, BmAtg3, and BmAtg8 in *Bombyx mori* using western blotting (A) and immunofluorescence staining in fat body after treatment with BmAtg4 RNAi for 24 h (A’; Scale bar: 10 μm). Measuring the protein concentrations of BmSqstm1, BmAtg3, and BmAtg8 using western blotting in the fat body after treatment with BmAtg7 RNAi for 24 h (B). At the same time point, protein concentrations of BmAtg3 and BmAtg8 were studied using immunofluorescence staining (B’; Scale bar: 10 μm). Acetylated BmAtg4-V5 after 4-h starvation (C). Acetylated BmAtg7-HA concentrations after 4 h (D). BmAtg8, BmSqstm1, and BmAtg4-V5 concentrations were analyzed using western blotting (E) and immunofluorescence staining (E’; Scale bar: 10 μm) after treatment with 20-hydroxyecdysone (20E) or 4-h starvation. Protein contents of BmSqstm1, BmAtg7-HA, and BmAtg8 were assessed using western blotting (F), and immunofluorescence staining (F’; Scale bar: 10 μm) was used to detect BmAtg7-HA concentrations after treatment with 20E or 4-h starvation. Acetylated levels of BmAtg4-V5 were measured after treatment with 5 μM 20E, 800 nM C646 (selective inhibitor of P300), 20 μM TSA (deacetylase Rpd3 inhibitor), and 20 μM CTB (P300 activator) after 6 h in BmN cells (G). Acetylated levels of BmAtg7-HA in BmN cells after treatment with the same conditions mentioned in panel G (H). Adapted with permission [59]. Copyright Cell Death Discovery 2021 (https://doi.org/10.1038/s41420-021-00513-0). Atg, autophagy-related protein; TSA, Trichostatin A.

**FIGURE 3:**
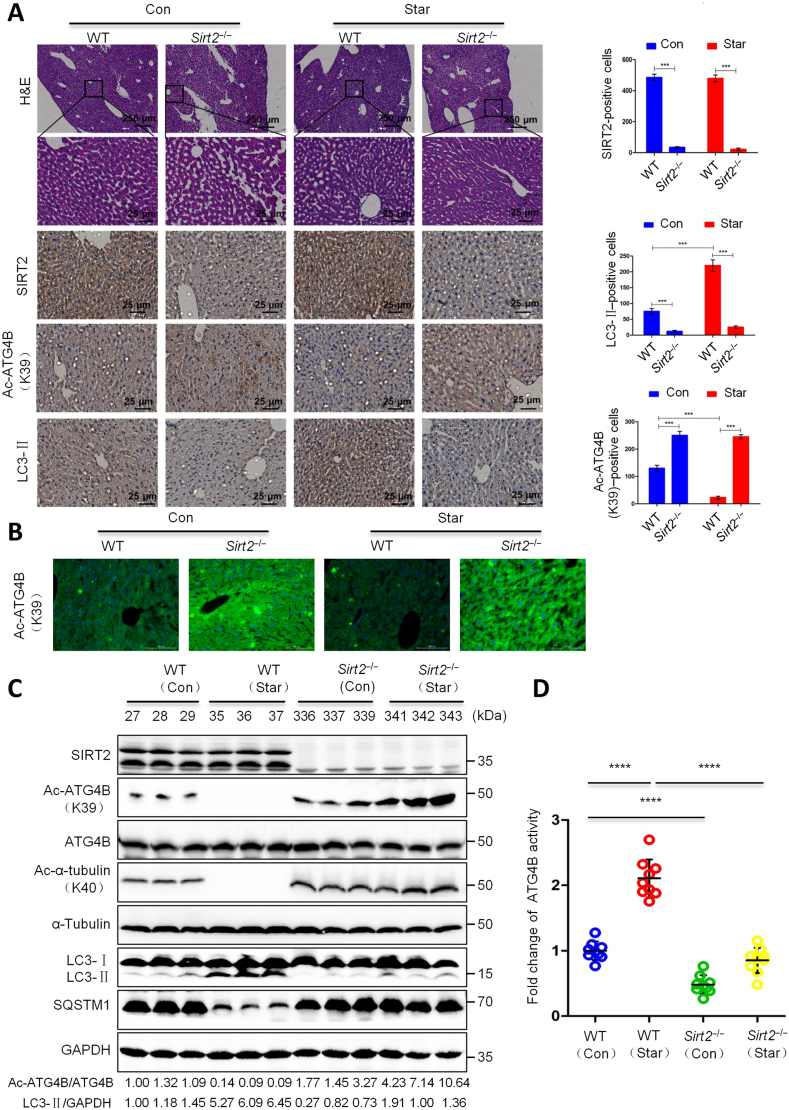
Studying the effect of SIRT2 on ATG4B deacetylation and autophagy regulation in in vivo conditions. *Sirt2*^*−/−*^ C57BL/6N mice were starved for 2 d, and hepatic tissue was analyzed and compared with the wild-type control group. Immunohistochemical analysis of SIRT2, Ac-ATG4B^K39^, and LC3 (A). Positive cells were counted and calculated per 1000 cells. Ac-ATG4B^K39^ concentrations were analyzed using immunofluorescence staining (B; Scale bar: 25 μm). Measuring protein concentrations of Ac-ATG4B^K39^, Ac-α-tubulin^K40^ using western blotting (C). The activity of ATG4B (D). The analyses were done in triplicate. One-factor ANOVA with Tukey’s multiple comparisons tests. ∗∗∗*P* < 0.001; and ∗∗∗∗*P* < 0.0001. Adapted with permission [58]. Copyright Science Advances 2022 (https://doi.org/10.1126/sciadv.abo0412). ATG, autophagy-related protein; SIRT2, sirtuin 2.

Emerging data have indicated a close relationship between IF and autophagic response. How and by which mechanisms IF can regulate the autophagy machinery is the subject of debate [57]. Sun and coworkers [58] found a close relationship between starvation and autophagy induction via the deacetylation of ATG4B and further interaction with pro-LC3. It confirmed that starvation in mice led to simultaneous reduction of P300 and induction of SIRT2 activity, resulting in deacetylation of ATG4B [58]. Under normal conditions, the acetyltransferase activity of P300 contributes to the nuclear localization of the ATG8–PE ubiquitin system and autophagy inhibition in *Bombyx mori* (Figure 2) [59]. Based on the data, a 2-d period of starvation in *Sirt2*^*−/−*^ mice led to a reduction of LC3-II, an increase in acetylated ATGB4 in hepatic tissue, and a suppression of the autophagic response, suggesting a close relationship between autophagy and SIRT2 activity (Figure 3) [58]. Once the intracellular concentrations of ATP and glucose drop below the normal values, the elevation of AMP promotes the allosteric changes in AMPK and thus its enzymatic activity [60]. In such conditions, the AMPK activity inhibits mTORC1 and protein synthesis to minimize ATP consumption by controlling anabolic and catabolic processes [61]. Further reduction of fructose-1, 6-bisphosphate concentrations activate lysosomal AMPK via vacuolar H^+^‒ATPase-aldolase complex. Along with these changes, the AXIN-vacuolar H^+^‒ATPase complex regulates the activity of LKB1, resulting in the phosphorylation of AMPK [61]. To minimize fatty acid synthesis, AMPK recalls acetyl-CoA carboxylases to trigger the oxidation of fatty acids. Furthermore, the phosphorylation of SREBP-1c by AMPK leads to the suppression of fatty acid synthesis at the expression level [61]. Direct phosphorylation of ULK1 (the ortholog of yeast Atg1) and BCLN1 by AMPK initiates several Atgs related to the autophagy signaling pathway [62].

### Regulation of autophagy by insulin signaling

The insulin signaling pathway has a critical role in the regulation of anabolic and catabolic mechanisms under physiological and pathological conditions [63]. Insulin hormone can bind to membrane-associated receptors INSR-1, -2, and IGF1R, which have tyrosine kinase activity, to trigger signals associated with energy homeostasis [64]. Unlike hypoglycemic conditions, the activation of INSR-1, -2, and IGF1R increase glucose uptake via the PI3K/Akt axis [63,64]. Along with these changes, glycolysis, glycogenesis, and lipogenesis mechanisms are induced by the activation of phosphofructokinase‒2 and inhibition of GSK3β [63,64]. Gluconeogenesis is reduced in the presence of insulin following the phosphorylation of FOXO1 [65,66]. Data support the fact that insulin inhibits the autophagy machinery via the activation of mTORC1, phosphorylation of ULK1, and suppression of FOXO1 expression [67]. Recent works have reported that Akt regulates the phosphorylation of mTORC1 and FOXO1/3 factors, indicating the reciprocal interaction of autophagy and insulin signaling pathways [67]. Results showed a moderate to high increase of aging-related factors like p53, p21, and p16 and β-galactosidase activity in chondrocytes exposed to hyperglycemic conditions [68]. By increasing the glucose contents, intracellular p62 and ROS concentrations are increased, and this coincides with the suppression of LC3II/I and the phosphorylated-AMPK/AMPK ratio [68]. Of course, it should not be forgotten that the autophagy machinery is stimulated in the early stages after the exposure of cells to high glucose conditions. However, it is possible that the continuity of hyperglycemic conditions leads to an abnormal (excessive) autophagic response [9,69]. Taken together, these data suggested the presence of common effectors between the glucose metabolism, insulin signaling pathway, and autophagy response and their mutual interaction under several conditions.

### Role of IF on Autophagic Response under Pathological Conditions

The apparent and early-stage activity of the autophagy machinery has been indicated in several pathologies [70]. In line with this claim, evidence for the participation of autophagy as an early-stage protective response has been obtained in different neurodegenerative diseases like Huntington’s disease, AD, Parkinson's disease, etc. [71]. With the progression of neurodegenerative disease, the accumulation of misfolded proteins and peptides increases the possibility of proteotoxicity [72,73]. The physiological significance of autophagy is related to scavenging deleterious proteins and the prohibition of neuronal proteotoxicity [74]. Commensurate with these descriptions, it is thought that autophagy activation using diverse stimuli like regular food starvation and varied exogenous compounds is a therapeutic strategy in patients [75,76]. An experiment conducted by Alirezaei et al. [76] confirmed that short-term fasting (24–48 h) induces an autophagic response in murine hepatic tissues and neuronal cells. Based on the data, the number of hepatic cell autophagosomes (evidenced as GFP-LC3 puncta) was increased in the early 24 h post-food restriction, and these values reached maximum concentrations after 48 h [76]. A similar pattern was obtained in the cortical neurons of food-restricted mice compared with those of the control group. Both the number and size of autophagosomes were increased in food-restricted mice. Interestingly, these conditions can promote retrograde traffic of autophagosomes from neurites toward cell somata, indicating the formation of autophagolysosomes and lysosomal degradation [76]. Likewise, the existence of reticular perinuclear patterns in Purkinje cells indicated active autophagosome-lysosome fusion after food restriction [76]. As such, it was suggested that reduced p70S6 kinase activity and phosphorylated S6RP in nonfed mice were associated with the inhibition of mTOR and the autophagic response [76]. These data show that food restriction can improve neuronal injury associated with proteotoxicity and changes in intracellular inclusion via the lysosomal degradation system. In support of this notion, SIRT1-mediated deacetylation of FoxO affects the activity of ROCK1, which in turn promotes α-secretase and reduces Aβ deposition [77]. It has been shown that α-secretase, a metalloproteinase, can degrade amyloid precursor protein within the Aβ domain and inhibits the accumulation of insoluble Aβ in the extracellular matrix [78]. These data indicate the potential clinical relevance and neuroprotective effects of calorie restriction on neurological disorders via the modulation of autophagy. Toxic protein aggregates can be removed via therapeutics (drugs) or certain food regimes that target autophagy machinery. Because calorie restriction is an inexpensive and suitable alternate to drug administration and available medications, it seems that calorie restriction can be used as a prophylactic and therapeutic approach in patients with neurological disorders. Despite the beneficial effects of food restriction in the alleviation of neurodegenerative disease, there are some conflicting and inconsistent results [79,80]. Every-other-day feeding of 5XFAD mice (AD model) not only did not reduce plaque formation in brain parenchyma but also promoted a cortical neuroinflammatory response and reduced protein concentrations of factors associated with synaptic plasticity [80]. This apparent discrepancy may be related to several factors, such as food components, experimental protocol, period of dietary restrictions, genetic traits, and age of studied groups [81]. A plethora of documents have indicated compromised autophagy flux and reduced lysosomal proteolysis under calorie restriction conditions [82]. That said, calorie restriction in the elderly may not be a good strategy to eliminate the intracellular inclusion bodies because of the compromised autophagy machinery. Furthermore, cells can adapt themselves with regulated energy reduction, acute depletion in a short time, or severe ATP reduction in a prolonged time, which inhibit vacuolar hydrolysis. Under these circumstances, macroautophagy is integral to the progression of pathological changes [83]. Even the significant and hostile depletion of ATP forces the cells toward necrotic changes rather than apoptosis and/or autophagic death [84]. Rapid ATP depletion activates excessive autophagy flux, which exceeds the degradation capacity of lysosomes, resulting in the accumulation of autophagosomes and cell death [8,85]. It should not be forgotten that some treatment protocols are not applicable in in vivo conditions with prolonged fasting. For example, in rats subjected to overnight fasting, local brain calcium contents were abnormally increased after systemic administration in which refeeding with glucose led to return of brain calcium to normal concentrations [86]. One reason for these effects may be that chronic starvation can lead to blood-brain-barrier leakage due to rapid ATP depletion and loss of cell homeostasis. Thus, attention should be given when certain starvation protocols are suggested for patients with conventional therapeutic modalities.

Like the central nervous system, autophagy flux has a crucial role in heart function and cardiomyocyte homeostasis [87]. The activation of AMPK in ischemic cardiomyocytes is a housekeeping protective mechanism to save the injured cells [88,89]. IF in ECs mediates functional activity and reduces injury rates in obese individuals by regulating certain biomarkers and the autophagic response [90]. Godar and coworkers [91] demonstrated the cardioprotective role of IF on ischemia-reperfusion injury in a mouse model. Six-week alternate-day fasting increased autophagy flux (autophagosomes**↑**) and reduced infarct area compared to nonfasted mice. They claimed that the inhibition of autophagosome-lysosome fusion using chloroquine led to the accumulation of LC3-II and SQSTM1/p62 [91]. Suppression of lysosomal *Lamp2* increased punctate GFP-LC3 and SQSTM1/p62 such that IF worsens ventricular remodeling and cardiomyocyte toxicity in *Lamp2*^*-/-*^ mice [91]. These features indicate that the activation of the lysosomal degradative machinery via autophagy following IF can exert regenerative outcomes in cardiac tissue with the occurrence of ischemic changes. Of note, active Atg5 expression and autophagic flux in starved cardiomyocytes contribute to resistance against ischemic conditions [92]. In aged rats subjected to acute myocardial infarction with isoproterenol, 4-wk IF led to upregulation of Atg5 and reduction of cardiac creatine kinase, MDA, TNF-α, and FBS [92]. Within hepatic tissue, autophagy function is very important due to its unique metabolic status and specific function [93]. Therefore, it is logical to hypothesize that slightly insulting conditions and metabolic disorders can lead to the activation of autophagy inside hepatocytes [94]. A deficient autophagic response can lead to the progression of several pathologies such as nonalcoholic steatohepatitis, viral diseases, and varied carcinomas [93]. It was suggested that 72 h fasting per week for 8 wk can reduce fat mass in mouse liver fed with a high-fat diet by the induction of LC3, LAMP1, and BCLN1 compared to the feed pellets–fed mice [57]. No significant differences were found in terms of autophagy status in the skeletal muscles [57]. One reason would be that the liver is the primary site for the production and accumulation of ketone bodies during starvation and there is a close relationship between autophagy status and ketone body concentrations [95]. The downregulation of Atg7 is associated with the intracellular accumulation of NCoR1, a PPAR-α coregulator, inhibition of PPAR, and reduction of fatty acid β-oxidation [96]. Therefore, the sensitivity of tissue to IF can be different, with varied autophagic responses [57]. Of note, IF integrates a multiplicity of signaling effectors to activate adaptive autophagy in response to ischemic conditions [97]. In fasted mice subjected to hepatic tissue ischemic reperfusion injury, time-dependent protection following IF should not be neglected [97]. The transcription of proinflammatory genes such as *Tnfα*, *Il1b*, *Il6,* and *Ccl2* and increased systemic HMGB1 concentrations were found to be mitigated in 1-d fasted mice with injured hepatocytes, whereas 2- and 3-d fasting protocols did not exert protective effects [97]. These effects are associated with the regulation of resident Kupffer cells within the hepatic tissue and neutrophil infiltration in response to ischemic injury. Along with these changes, the production of ROS and TNF-α was not increased in LPS-treated mouse Raw 264-7 macrophages exposed to decreasing concentration of fetal bovine serum [97]. Data indicated the increase of LC3, BCLN1, SIRT1, and intracellular aggregation of HMGB1 in the fasted mice as compared to the control-matched groups [97]. Autophagy machinery is thought to degrade proinflammatory HMGB1 and prohibits further secretion into the blood and activation of phagocytes [97,98]. Therefore, one can hypothesize that regular fasting can activate the autophagic response in ischemic hepatocytes, thus permitting direct degradation of proinflammatory factors and reducing their release into the extracellular matrix. In a comparable study, Chen and colleagues [99] extended our understanding of the potential impact of IF on hormonal regulation of lysosomal degradation capacity. They observed the elevation of systemic FGF21,which can elevate fatty acid β-oxidation rate, and autophagic response (GFP-LC3 puncta↑) in mice subjected to 12- and 24-h fasting protocols [99]. Using *Fgf21*^*−/−*^ mice, it was indicated that intracellular LC3-II and p62 concentrations were significantly elevated, coincident with the inhibition of *Lamp1* and the lysosomal-autophagic pathway [99]. In wild-type mice, fasting can inhibit mTORC1, leading to the reduction of phosphorylated TFEB factor and sequestration inside the cytosol. Dephosphorylated TFEB translocates into the nucleus and stimulates autophagy and lysosomal activity. It is noteworthy to mention that the ablation of *Fgf21* can increase the activity of Mid1 and phosphorylation of TFEB. Given the intricate connection between fasting and lipid metabolism, it is likely that IF can regulate autophagic flux via the regulation of lysosomal function. Recently, it was suggested that calorie restriction along with the stimulation of autophagy can change the type of cell death and reduce consequent pathological outcomes [100]. It was indicated that the promotion of autophagy via rapamycin can reduce the severity of caerulein-induced acute pancreatitis in mice via the increase of Caspase-3 (an apoptosis-related factor) and reduction of HMGB1 (a necrosis-related factor). Amylase activity and systemic concentrations of proinflammatory factors such as IL-6, TNF-α, MCP-1, and GM-CSF were also diminished.

Myocytes differ fundamentally from other cells due to the presence of a specific system that provides energy in stressful conditions via the conversion of protein to amino acids [101]. Therefore, autophagolysosomes are basic degradation sites for the production of essential amino acids [101]. Findings are consistent with the fact that fasting can contribute to phenylalanine release from skeletal tissue in an mTOR-dependent axis. Furthermore, fasting can suppress mTORC1 activity and thus reduce phosphorylated ULK1 and 4EBP1. These alterations are concurrent with a simultaneous increase of intracellular concentrations of LC3B, p62, and FOXO3a [102]. Data have indicated opposite effects in the presence of insulin in which this hormone can reverse these conditions and near-to-normal conditions.

## IF and autophagy in cancers

A crucial role of calorie restriction and IF in terms of cancer development and growth has been indicated in numerous experiments [103,104]. On this basis, several forms of calorie restrictions such as dietary restriction, IF, and caloric restriction tout court have been applied to control the development and progression of various cancer types. Furthermore, the “caloric restriction mimetics” with limited calories provide calorie restriction-like conditions without the necessity for strict diets in cancer patients [105]. Several pieces of evidence revealed that low energetic diets can affect tumor cells’ metabolism and microenvironment per se, leading to a reduction of growth and metastasis rate [105]. Of note, diets with low carbohydrate and protein contents and high-fat content have several benefits in cancer patients in terms of survival rate and tolerating chemotherapy side effects [106]. It is believed that amino acids such as methionine, leucine, glutamine, arginine etc., as well as glucose have pivotal roles in dynamic growth of tumor cells and motility related to normal cell type. Thus, these features make cancer cells as frontline cells exposed to calorie restriction and starvation [105]. However, the exact impact of IF on anaplastic conditions remains greatly unknown and is under intense investigation [107,108]. Of course, calorie restriction cannot be an authoritative cancer treatment and is thought of as a supportive method to increase the therapeutic efficiency of chemotherapeutics [109] even though several studies in animal models have revealed the cancer-preventative relevance of IF [110]. Due to the inevitable role of IF on dynamic tumor cell growth, recent clinical studies have focused on the application of formulated and tolerable diets with certain fasting protocols in cancer patients [[111], [112], [113]]. Clinical observations have uncovered insulin and selected compounds such as IGF1 and glucose are reduced in individuals subjected to a 5-d fasting-mimicking diet [114]. Such a metabolic profile can lead to the reduction of cancer rate, obesity, and inflammatory conditions in a mouse model [115]. As aforementioned, IF and calorie restriction can provoke autophagy signaling after cell exposure to insulting conditions, leading to the deactivation of mTORC1 [[116], [117], [118]]. Anti-tumor mechanisms such as those exerted by E-cadherin, the adiponectin/leptin ratio, metalloproteinases, chemokines and cytokines, Caspase-3, and Histone H2AX (an enzyme associated with DNA repair) are induced under starvation [[119], [120], [121], [122], [123]]. Furthermore, in vitro and in vivo starvation protocols can adjust the host cell’s sensitivity to insulin [124].

Preclinical data have indicated that short-period caloric restriction in cancer patients before or during the radiation course can increase tumoricidal properties [125]. Interestingly, Icard and coworkers [125] claimed that nonisocaloric ketogenic diets can contribute to conflicting effects in patients undergoing calorie restriction and radiotherapy compared with patients fed isocaloric diets. In vitro analyses indicated that the culture of several cancer cells such as HepG2 [hepatocellular carcinoma] and HuH6-clone5 [hepatoblastoma cells] in serum-free medium for 6 to 24 h increased radiosensitivity via the activation of mTOR and accumulation of ROS [126]. The exposure of human A549 cells to hypoglycemic conditions (2.8 mmol/L) for 24 h reduced clonogenic properties and increased DNA fragmentation after radiation, whereas normal HSF7 fibroblasts exhibited no sensitivity to glucose deprivation [127]. One reason may be that the basal metabolism status is carefully controlled by suppressive control checkpoints like SIRT3, PTEN, etc. in response to energy stress, making the normal cells flexible, whereas these factors are commonly inactive in cancer cells [128]. Of note, it seems the type of diet and period of calorie restriction can affect the dynamic growth of tumor cells [125]. Despite the stimulatory effect of ketogenic diets on the activation of autophagy response and healing mechanisms [129], the use of calorie restriction and therapeutic regimes in cancer patients with ketogenic diets can result in more conflicting outcomes [125]. In most cancer cells, protein concentrations of enzymes catabolizing ketone bodies such as SCOT and 3β-OHBD are reduced compared to normal cells [130], and ketogenic diets are used for the prevention of weight loss in cancer patients undergoing therapeutic medications [131]. It was suggested that specific tumor cells from different tissues such as blood, breast, brain, etc. can promote the activity of SCOT and 3β-OHBD enzymes to catabolize fatty acids to increase tumor size. Thus, the application of unintended calorie restriction in cancer patients subjected to therapeutic protocols may lead to additional confounding bias [132]. In an experiment, 6-h fasting in rats fed with a ketogenic diet for 4 wk led to the reduction of LC3II and ULK and increase of serum creatinine and renal fibrosis [133]. High concentrations of triglycerides and cholesterol can affect the fusion of autophagosomes with lysosomes, resulting in the accumulation of autophagosomes and incomplete autophagic response. Besides these effects, excessive lipid intake with insufficient glucose can lead to abnormal glycolipid protein metabolism and the possibility of vascular tissue complications [133].

The activation of autophagy in cancer cells after IF may exert both detrimental and beneficial outcomes. The autophagy machinery prohibits cellular damage by the promotion of DNA repair systems and antioxidant mechanisms, whereas it accelerates tumor cell death under specific conditions. Interestingly, the activation of autophagy response in certain cancer cells is also associated with proliferation and metastasis [134,135]. These data indicate the double-edged sword activity of autophagy in terms of tumor dynamic growth. Whether and how autophagy orchestrates the protective and inhibitory mechanisms inside cancer cells should be elucidated. The suppression of BCLN1 is related to the possibility of varied cancer types in tissues such as the ovaries, breasts, and testes. The ablation of BCLN1 in mice led to the development of hepatic and pulmonary cancers because of a partially deficient autophagic response [116]. Of note, BCLN1 and BRCA1 (a tumor suppressor gene) are physically close to each other, and deletion of BRCA1 in breast cancer patients can affect BCLN1 [136]. Interestingly, certain tumor cells like several Ras-transformed anaplastic changes in the bladder, pancreas, colon, etc. exhibit stimulated autophagic responses related to normal cells. Therefore, modulation of autophagy is a strategic approach to the control of these cancer types [137]. The promotion of the GTPase KRAS is tightly correlated with autophagy. Of note, in some tumor cells, mutation of Ras genes (H-RasV12 or K-RasV12) can lead to elevated autophagic flux despite the activation of mTORC1 [137]. It has been shown that reduced calorie intake can make tumor cells susceptible to apoptosis via the stimulation of CD8 lymphocytes [138]. Under fasting conditions, the inhibition of mTORC1 (AMP/ATP↑) can lead to the activation of autophagy, promotion of ketogenesis, and inhibition of glycolysis and glutaminolysis in tumor cells [139]. Calorie restriction can diminish serum concentrations of IGF-1 with the potential to suppress the PI3K/Akt/mTOR axis and thus glycolysis in cancer cells [139]. Of note, the reduction of glycolytic capacity in cancer cells is integral to lowering the proliferation rate [140]. Along with these changes, the differentiation rate toward Th1, Th17 lymphocytes and M1 macrophages is increased [139]. IF may reduce the density of CD3^+^/CD4^+^/CD25^+^ T regulatory cells within the tumor parenchyma [141]. In response to calorie restriction, the sensitivity of natural killer cells to tumor cells is also heightened.

**FIGURE 4:**
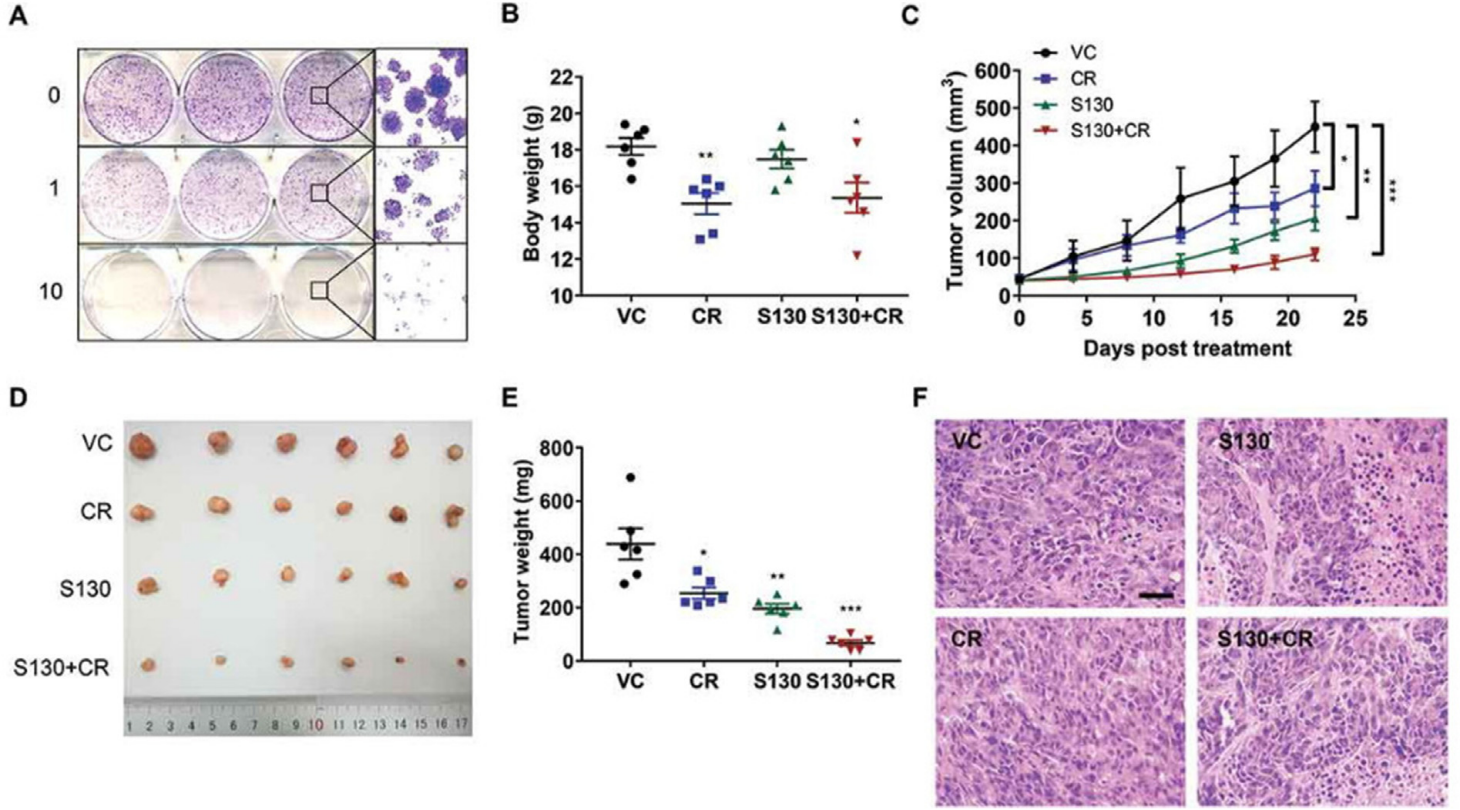
The role of ATG4B inhibitor, S130, on the dynamic growth of colorectal cancer cells (A–F). HeLa cells were incubated with 1 and 25 μM of S130 and clonogenic properties were assessed using crystal violet staining (A). Panels B–E indicate the combined effects of S130 (i.p. 20 mg/kg) and calorie restriction on the body weights (B), tumor volume (C), tumor images, and weights (D–E) in mice with HCT116 xenografts. Hematoxylin–eosin staining of tumor mass in different groups (F). Student 2-tailed *t* test. Data are expressed as mean ± SEM (*n* = 6). ∗*P* < 0.05; ∗∗*P* < 0.01; and ∗∗∗*P* < 0.001 relative to VC. Mice were subjected to calorie restriction with 70% food intake. Adapted with permission [144]. Copyright Autophagy 2019 (https://doi.org/10.1080/15548627.2018.1517073). ATG, autophagy-related protein; CR, calorie restriction; VC, vehicle.

Of note, the induction of certain oncogenes such as PI3K, Akt1, and Bcl2 suppresses the autophagic response. The downregulation of varied proteins (DAPK, PTEN, TSC1, TSC2, and LKB1/STK11) is associated with lysosomal degradation capacity in cancer cells [142]. Upon exposure to calorie restriction and lack of sufficient ATP, cells try to adapt themselves to insulting conditions (ie, chemotherapy) via the activation of autophagy, which is not activated in cancer cells, and therefore predisposes them to cell injury [143]. In an experiment, the effect of calorie restriction (70% of normal food intake) was investigated in the dynamic growth activity of human colorectal HCT116 tumor cells in a mouse xenograft model [144]. It was suggested that inhibition of ATG4B cysteine protease activity using S130 along with calorie restriction led to the accumulation of LC3-II (increased delipidated LC3) and p62 inside the cancer cells in tumor-bearing mice, resulting in apoptotic death via Caspase-3 activity (Figure 4) [144]. In vitro analysis indicated that the culture of MCF-7 and HCT116 cells under calorie restriction conditions led to the upregulation and activation of protein kinase CK2 (isoforms CK2α and CK2β), SIRT1, and phosphorylated AMPK [145]. These features coincide with the activation of autophagy molecular machinery (Atg5, Atg7, LC3BII, BCLIN1, and Ulk1). Calorie restriction can intensify the inhibitory effect of siRNA on protein kinase CK2 and inhibition of autophagic response in these cancer cell lines [145]. Data confirmed that the growth of Atg5^+/+^ kidney epithelial cells was reduced in nude mice fed with a calorie-restriction diet compared to the group fed with a standard regime [146]. In this regard, Lashinger and coworkers [146] claimed that the reduction of systemic glucose and amino acids and the concurrent increase of ketone bodies can abrogate the normal autophagic flux. Interestingly, the dynamic growth of Atg5^-/-^ kidney epithelial cells was profoundly reduced in situations exposed to calorie restriction. These data showed that the modulation of autophagy along with calorie restriction can affect the expansion of tumor cells in in vivo conditions. Although calorie restriction can reduce the proliferation of Atg5-expressing cancer cells, these effects were intensified with simultaneous inhibition of the autophagy molecular machinery. Sun and colleagues [147] demonstrated that alternate-day fasting for 14 d can exert tumoricidal effects in the murine colorectal carcinoma CT26 cell line. Data indicated that tumor size and M2 polarization of tumor-associated macrophages were reduced under restricted calorie conditions. Along with the induction of autophagy (Atg5**↑**, and LC3II/I ratio**↑**) in fasting mice, the expression of CD73 and adenosine production were also diminished [147]. Recently, it has been indicated that induction of excessive ROS and nitrogen species production using cold atmospheric pressure plasma led to autophagic and apoptotic death in B16 melanoma cells after being inoculated subcutaneously in mice [148]. Starvation of mice for 24 h (3 times per 5 d) led to prominent tumoricidal effects compared to mice subjected to cold atmospheric pressure plasma. Based on the data, excessive autophagy response (LC3↑, and ATG5↑) initiated apoptotic changes that coincided with the activation of proapoptotic factors such as Bax and Caspase-3 [148]. The overactivity of Atg5 promotes its cleavage by calpain and further interaction with Bcl-X, resulting in apoptosis [149].

**FIGURE 5:**
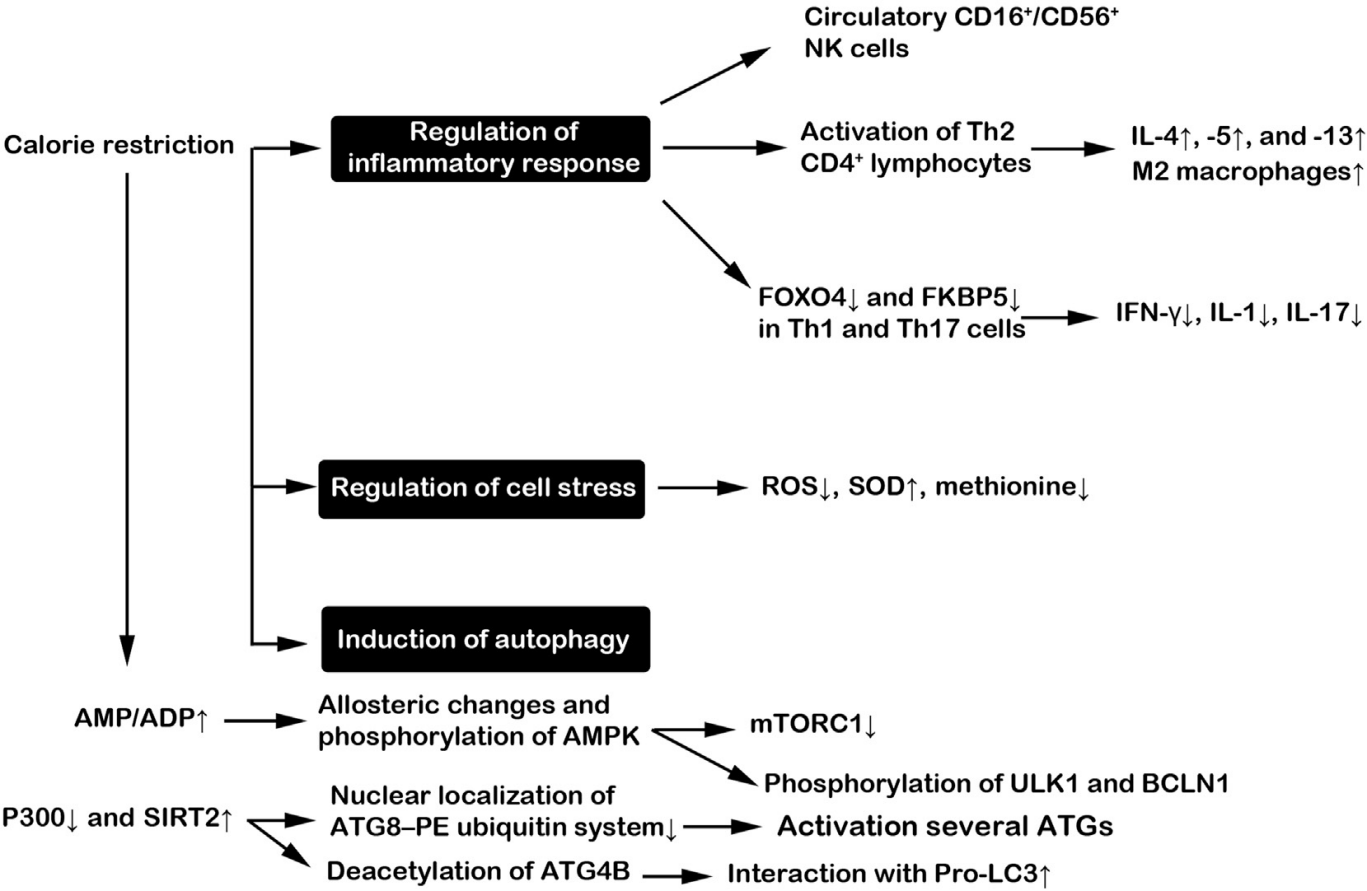
Possible effects of calorie restriction on autophagic response, cell stress, and immune cell activity.

In conclusion, in this review, the protective/detrimental effects of calorie restriction-modulated autophagy were discussed. Data support the fact that adaptive and excessive autophagy can be activated depending on the intensity and duration of calorie restriction programs. It seems that controlled calorie restriction or IF can exert protection on different cellular functions by a modulation of autophagy in response to several pathological conditions (Figure 5). By the activation of adaptive autophagy and relevant downstream effects, IF or calorie restriction can lead to lifespan-extending and antiaging effects via the regulation of adequate homeostasis. Moderate calorie restriction for short- and long-term or IF practiced in various religions in young and middle-aged individuals can be used with no prominent adverse effects [150]. Despite the advantages, insufficient energy intake and dehydration are the risk factors in volunteers with calorie restrictions that should be carefully monitored. Under these conditions, defective (excessive) autophagy response not only does protect the host cells but also forces the cells toward autophagic cell death and apoptosis. Numerous investigations are mandatory to address underlying mechanisms related to autophagy modulation in individuals practicing IF. Despite the modulatory effects of calorie restriction on the autophagy signaling pathway, the underlying mechanisms associated with the activation of adaptive/excessive autophagy have not been addressed. The advances in molecular biology will enable us to monitor real-time changes in autophagy response in individuals under different IF programs. Due to the lack of sufficient data associated with the safety and side effects of IF programs in patients, calorie restriction should be used in clinical settings with great caution. The combination of autophagy inducers and certain calorie restrictions under specific pathological conditions can help clinicians to develop de novo therapeutic protocols.

## Acknowledgments

We thank the personnel of the Infectious and Tropical Diseases Research Center for their help and guidance.

### Author contributions

The authors’ responsibilities were as follows—RS, SH, MSM, AB, FS, HSB: conducted the literature search, extracted the data, and drafted the relative data parts; RR, ES: supervised the study; and all authors: read and approved the final manuscript.

### Conflict of interest

The authors report no conflicts of interest.

### Funding

This is a report of the database from an MSc thesis registered in Tabriz University of Medical Sciences with the Number 67863 (Ethical code: IR.TBZMED.VCR.REC.1401.11).
